# The node of Ranvier in CNS pathology

**DOI:** 10.1007/s00401-014-1305-z

**Published:** 2014-06-10

**Authors:** I. Lorena Arancibia-Carcamo, David Attwell

**Affiliations:** Department of Neuroscience, Physiology and Pharmacology, University College London, London, WC1E 6BT UK

**Keywords:** Neuron, Node of Ranvier, Sodium channel, Myelin

## Abstract

Healthy nodes of Ranvier are crucial for action potential propagation along myelinated axons, both in the central and in the peripheral nervous system. Surprisingly, the node of Ranvier has often been neglected when describing CNS disorders, with most pathologies classified simply as being due to neuronal defects in the grey matter or due to oligodendrocyte damage in the white matter. However, recent studies have highlighted changes that occur in pathological conditions at the node of Ranvier, and at the associated paranodal and juxtaparanodal regions where neurons and myelinating glial cells interact. Lengthening of the node of Ranvier, failure of the electrically resistive seal between the myelin and the axon at the paranode, and retraction of myelin to expose voltage-gated K^+^ channels in the juxtaparanode, may contribute to altering the function of myelinated axons in a wide range of diseases, including stroke, spinal cord injury and multiple sclerosis. Here, we review the principles by which the node of Ranvier operates and its molecular structure, and thus explain how defects at the node and paranode contribute to neurological disorders.

## Introduction

Timely delivery of information is essential for the proper function of the nervous system. The increase in axonal conduction speed produced by myelination allows rapid transmission of electrical signals over large distances between neuronal cell bodies and their axon terminals, and thus confers improved cognitive function [44]. Precise regulation of this conduction velocity may allow tuning of motor skills and sensory integration [128, 147]. Consequently, any disruption, in pathology, of the two specialised structures that are essential for rapid conduction—the myelin sheath and the node of Ranvier—can be expected to alter sensory perception, cognitive processing and motor output. Numerous articles have reviewed the malfunctions which stem from loss of the myelin sheath in disorders such as multiple sclerosis, stroke, spinal cord injury and cerebral palsy [44, 94, 95, 134, 146]. However, despite its importance, pathological disturbances of the node of Ranvier (and its surrounding domains) have received far less attention. Here we aim to redress this situation (see also [139]), because recent studies have highlighted structural changes at the node of Ranvier and altered function of proteins at this site as key players in neuronal dysfunction.

## Myelin and the node of Ranvier

Saltatory conduction along myelinated axons evolved in vertebrates as a way of rapidly transmitting electrical impulses across large distances. The axon down which the action potential travels is wrapped by myelin, which is produced by oligodendrocytes in the central nervous system (CNS) and by Schwann cells in the peripheral nervous system (PNS). This myelin sheath increases the effective resistance of the axonal membrane, lengthening its electrical space constant and thus promoting signal spread along the axon. More importantly [7], however, myelin decreases the effective capacitance of the axonal membrane, so that less charge (in the form of Na^+^) needs to enter to depolarize the cell. Both of these effects increase the action potential conduction speed. In addition, the reduction of Na^+^ entry leads to less ATP being used by the axon on Na^+^ pumping, thereby allowing the conduction of the action potential to be more energetically efficient for the axon, at the expense of more energy being used to maintain the resting potential of the ensheathing oligodendrocyte [55].

**Fig. 1:**
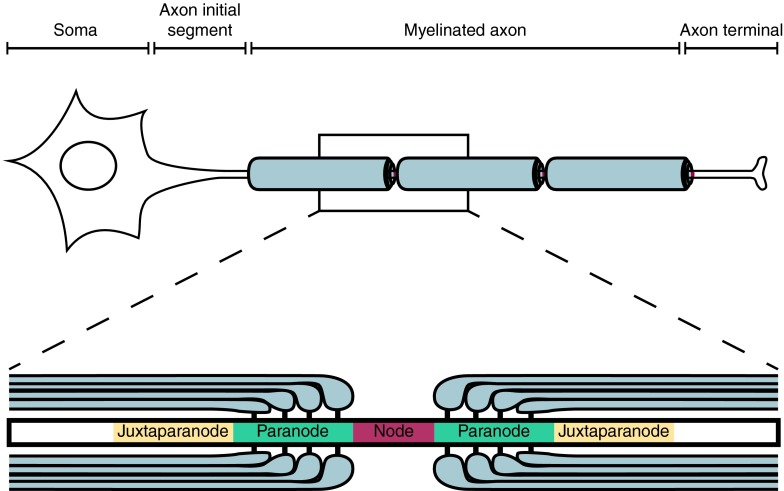
Schematic diagram showing the different domains of a myelinated neuron. The axonal region around the node of Ranvier is expanded to show the different axonal domains: the node of Ranvier where voltage-gated Na^+^ channels are expressed, the paranode where the myelin is attached to the axon, and the juxtaparanode where most voltage-gated K^+^ channels are located. Each of these domains is characterised by the expression of specific proteins (shown in Fig. 2)

**Fig. 2:**
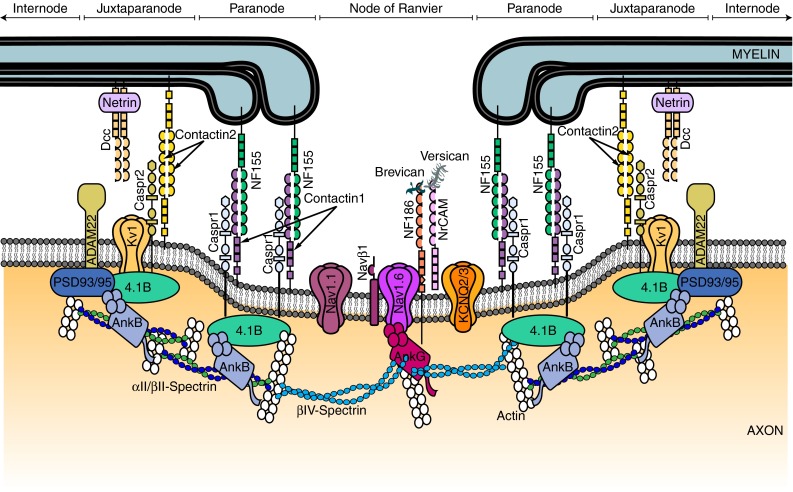
Schematic diagram of the proteins at the node of Ranvier, paranode and juxtaparanode. These domains are the location of ion channels (Nav1.6 and Nav1.1, KCNQ2/3, Kv3.1 and Kv1.1/1.2), cell adhesion molecules (neurofascin 155 (NF155), neurofascin 186 (NF186), contactin 1 and 2, contactin-associated protein (Caspr 1 and 2), cytoskeletal scaffolding proteins [Ankyrin (Ank) G and B, protein 4.1B, and postsynaptic density protein 93/95 (PSD93/95)], cytoskeletal proteins (βII- and βIV-spectrin), and extracellular matrix proteins (brevican, versican and a secreted form of NrCAM). Targeting and scaffolding mechanisms ensure that each protein is segregated to its specific subdomain

Fundamental to the function of myelinated axons is the existence of discrete sites where Na^+^ enters to generate the action potential: the nodes of Ranvier are small (~1 μm long) regions along myelinated axons where there is a break in the myelin sheath and the axon membrane is in contact with the bulk extracellular space, allowing Na^+^ entry through voltage-gated channels (Fig. 1). In order for nodes to form and function correctly, highly complex interactions are needed between the axon and its ensheathing glial cell. These interactions have four functions. They serve to: (1) define the node as an area free of the glial sheath, (2) localise voltage-gated Na^+^ (Nav) channels in the axonal membrane at the node, (3) localise axonal K^+^ channels on either side of the node, and (4) attach the ends of the myelin sheaths to the axon on either side of the node so that current cannot easily pass under the myelin (which would negate its membrane capacitance-reducing and resistance-increasing effects). A large array of scaffolding and cell adhesion proteins is required to mediate these axon–glial interactions (Fig. 2), and the complexity of the protein–protein interactions involved makes the structure of the node and surrounding regions prone to disruption in pathological conditions.

To understand how nodal function may be disrupted in disease, we need to understand the electrical principles by which the node operates, how it is formed and the molecules which are essential for its function.

## Electrical principles for healthy node of Ranvier function

Four general principles must be obeyed if the node of Ranvier is to function correctly. Firstly, the molecular mechanisms described below must produce a high concentration of Nav channels in the nodal membrane, where they will experience the full transmembrane voltage change of the action potential. This is needed for rapid activation of the channels. Any channels that are mislocalised to internodal regions, where they are covered with multiple layers of myelin, will experience a smaller voltage change and activate less, and more slowly, because in the internode the voltage change of the action potential (~100 mV) will be spread across 2*N* + 1 membranes (where *N* is the number of myelin wraps), resulting in axonal channels experiencing a depolarization of only 100/(2*N* + 1) mV or around 9 mV for a typical CNS axon with *N* = 5 wraps. In addition to being localised at the node, there needs to be a sufficient number of Nav channels at the node to generate enough current to depolarise the next node along the axon: a decrease of the number of functioning Nav channels may cause propagation failure.

Secondly, most voltage-gated K^+^ channels (Kv1 type, see below) are localised to the juxtaparanodal region (Fig. 2) [47, 112], although Kv3.1b channels and slowly activating KCNQ (also known as Kv7) channels are present at the node itself [32, 34]. Placing K^+^ channels in the juxtaparanode under the myelin sheath will reduce the voltage they experience, and hence reduce their activation under normal conditions, for the reasons described above. However, the exact degree of activation occurring will depend on how much current flow can occur under the myelin to the juxtaparanodal region, to maintain the extra-axonal voltage close to the bulk extracellular value of 0 mV. The application of 4-aminopyridine (4-AP, which blocks Kv1 type channels) has little effect on the propagation of single action potentials (bursts of action potentials were not tested) in dorsal column axons [76] and similarly knockout of Kv1.1 produces only a small prolongation of the action potential in PNS axons [132]. However, more variable effects of 4-AP were reported in optic nerve axons, with Foster et al. [45] reporting relatively minor effects (compared to those on unmyelinated axons) while Devaux et al. [32, 33] found a profound broadening of the action potential, suggesting that these channels contribute different amounts to action potential repolarization in different axons. Although Kv3.1b channels are also blocked by 4-AP (unlike KCNQ channels), the action potential broadening that 4-AP produces in optic nerve axons is still seen with Kv3.1b knocked out [32] implying that 4-AP has its effect by acting on Kv1 channels. Furthermore, during node development, at least in the PNS, these channels prevent re-entrant excitation of the nodes following single impulses [148].

**Fig. 3:**
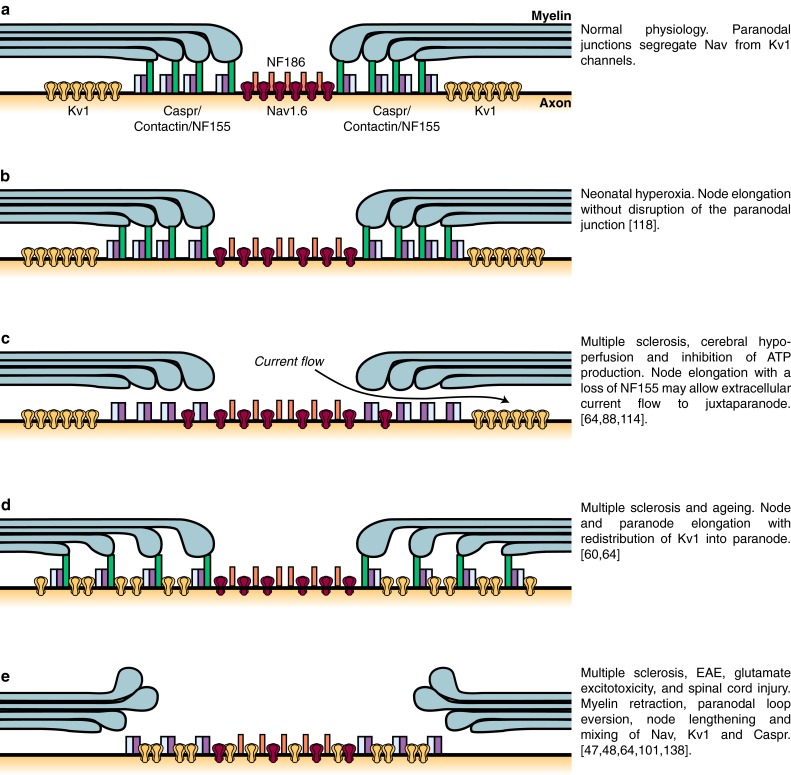
Cartoon illustrating potential mechanisms underlying enlongated nodes of Ranvier in different pathologies. **a** Diagram of the nodal region in control conditions. Nav channels and Kv1 channels are segregated by paranodes. **b** In neonatal hyperoxia, nodes of Ranvier are enlarged without any observed disruption of the paranodal junction. **c** In multiple sclerosis, cerebral hypoperfusion and in an experimental model of energy deprivation, the node of Ranvier is enlarged, there is a loss of NF155, and Nav channels slightly overlap with the paranodal protein Caspr. The disruption of the paranodal junction may allow current flow to under the myelin sheath to promote the activation of Kv1 channels at the juxtaparanode, thereby compromising action potential firing. **d** In the ageing brain and in multiple sclerosis, an elongation of the paranode can be caused by a separation of the paranodal loops. This is accompanied by a redistribution of Kv1 channels into the paranodal area, where they overlap with Caspr and NF155. **e** Myelin retraction, and a breakdown of the molecular organisation of the node of Ranvier, paranode and juxtaparanode, are observed in multiple sclerosis, EAE, glutamate excitotoxicity, and spinal cord injury

Thirdly, for the myelin to reduce the effective axonal membrane capacitance well, there needs to be little current flow under the myelin sheath [6, 120]. As described below in the description of the molecular apparatus at the paranode, this current flow is reduced by the formation of adhesive junctions between the ensheathing glial cells and the axon. However, these septate-type junctions still leave a spiral pathway between the extracellular space at the node and the extracellular space at the juxtaparanode [120], through which some current may flow to allow partial activation of juxtaparanodal K^+^ channels. During pathology, as described below, disruption of the paranodal structure may loosen this junction, leading to more extracellular current flow beneath the sheath (Fig. 3c) and more activation of the juxtaparanodal K^+^ channels, which may prevent action potential propagation, especially during repeated firing.

Fourthly, the nodal length and diameter need to be controlled. The speed with which nodal Nav channels activate is partly determined by the capacitance of the node, and hence by its membrane area [6, 153]. An increase in area, as occurs in some pathologies (see below), will tend to slow the action potential (unless extra Nav channels are inserted into the membrane to oppose this effect). Furthermore, an increase in node length, as also occurs in pathology, will increase the axial resistance for the current flow from the node to the internode that is needed to depolarise the next node, again slowing the action potential, or even causing it to fail.

Below, we will relate the molecular organisation of the nodal components, and the changes that occur in pathology, to these principles of nodal function.

## Molecular organisation of the node of Ranvier

As mentioned above, the node of Ranvier needs a high density of Nav channels to function correctly, and also has some Kv channels which (by controlling the resting potential and Nav channel inactivation) are thought to regulate the amount of current that the Nav channels generate [9]. In the CNS, the Nav channel isoforms found at nodes of Ranvier are Nav1.6, Nav1.1 and (earlier in development) Nav1.2 [35, 117]. Despite its small size (~1 μm), the node of Ranvier is a densely populated area of the axon, with a large number of cytoskeletal scaffold proteins and transmembrane adhesion proteins, all of which help to recruit and maintain the Navs at this site. PNS and CNS nodes have the same function, however, they differ slightly in structure and the mechanisms for their assembly are different [141, 158]. The biggest structural difference is that in the PNS, the nodes of Ranvier are apposed by nodal microvilli emanating from myelinating Schwann cells. These microvilli may play a role in local ion buffering, but also express gliomedin, a glial protein known to be important for the clustering of Nav channels at PNS nodes of Ranvier (for more information on PNS nodes of Ranvier see [13, 122]). Although CNS nodes of Ranvier are not contacted by protrusions of oligodendrocytes, they are often closely associated with astrocytic processes, the function of which remains unknown [58, 59].

The clustering of Nav channels at nodes of Ranvier is mediated by the scaffolding protein ankyrin G (Fig. 2). Ankyrin G is a large protein with various protein-binding domains that simultaneously connect a number of proteins at the node to the cytoskeleton (although ankyrin G has also been observed, albeit at much lower levels, localised to paranodes where it has an unknown role [111]). In addition to Nav channels ankyrin G has been shown to bind KCNQ channels, the adhesion protein neurofascin 186 (NF186) and cytoskeletal spectrins. This ability to bind the transmembrane protein NF186 and simultaneously bind the spectrin-based cytoskeleton is what allows ankyrin G to behave as a scaffold maintaining Nav and KCNQ channels at the nodes of Ranvier [and at the axon initial segment (AIS), where all these proteins are also found] [109, 110]. It has recently been shown that ankyrin G is transported down the axon to the nodes of Ranvier via a direct interaction with kinesin 1 [8]. Importantly, Nav channels are co-transported with ankyrin G, suggesting that ankyrin G is not only critical for the stabilisation of Navs at the node of Ranvier but also crucial for the correct subcellular targeting of these channels [8]. Ankyrin G is recruited to the nodes as a result of a direct interaction with NF186. NF186 plays an important role in the assembly and maintenance of the node of Ranvier via its interactions with the extracellular matrix components brevican, versican and a secreted soluble form of NrCAM, as well as via the aforementioned interaction with ankyrin G [31, 141]. NF186 may also play a role in the function of the node as it has recently been shown to be important for nodal expression of the Nav channel accessory beta1 subunit, which modulates Nav function [21, 31].

## Pathologies of the node of Ranvier

Although small changes at the node of Ranvier can have large effects on the speed of action potential propagation, it is only in the last decade that disruption of this site in pathology has been investigated in depth.

The correct function of nodal Nav1.6, Nav1.1 and KCNQ channels is important for saltatory conduction along myelinated axons. Alteration of the electrical excitability, permeability or expression of Nav1.6 or Nav1.1 channels can result in autism, epilepsy syndromes, periodic paralysis, or fibromyalgia (reviewed in [37]). Similarly, mutations in KCNQ2 and KCNQ3 channels result in benign familial neonatal convulsions and myokymia (spontaneous muscle quivering), highlighting their importance in controlling excitability [87]. However, given that these channels are not only expressed at CNS nodes, but also at the AIS and, to a lesser extent, somatodendritic compartments [34, 81, 84, 149], further work is needed to establish whether it is the defects in the function of these channels at the node of Ranvier that are responsible for these disorders.

Quivering 3J mice, which show a frame-shift base insertion in the Sptnb4 gene encoding βIV-spectrin and thus lack the C-terminus of βIV-spectrin, display neuromyotonic and myokymic discharges similar to those observed in mice with KCNQ2 mutations. Axons in the quivering 3J mice were properly myelinated and displayed proper targeting of Nav channels, Caspr and contactin, and Kv1 channels to the nodal and surrounding regions. However, despite KCNQ protein levels being unaffected overall in the brain, KCNQ channels were not found at either CNS or PNS axon initial segments or nodes of Ranvier, suggesting that the C terminal of βIV-spectrin is essential for correct localization of these channels to the Ranvier node and AIS.

An increase in the length of the node of Ranvier (defined by NaV or NF186 immunolabelling) has been observed in multiple sclerosis [64], experimental autoimmune encephalomyelitis (EAE) [47], ageing [60], cerebral hypoperfusion [114], inhibition of mitochondrial ATP production [88], spinal cord injury [101, 138], glutamate application to mimic its release in excitotoxic disorders [48], neonatal hyperoxia [118], and exposure to loud sounds [142] (Fig. 3). It has been suggested that this lengthening is due to myelin retraction and a breakdown of the paranodal junction (Fig. 3c, e), leading to a redistribution of Kv channels and current flow under the myelin sheath [48, 101] (see below). However, in some of these conditions it is possible that the node lengthens (giving the appearance of myelin retraction) whilst maintaining intact paranodal and juxtaparanodal structures (Fig. 3b) because there is an insertion of more membrane at the node, or lengthens due to a withdrawal from the axon of paranodal loops immediately adjacent to the nodal domains (adnodal paranodal loops). In this case, juxtaparanodal Kv channels may not be abnormally activated, but the increase of nodal surface area would lead to an increased nodal capacitance and, unless more Nav channels were inserted, a reduction of the action potential conduction velocity. A conduction velocity decrease has been observed in a number of transgenic mice in which some axons have normal nodal Nav channels, internodal myelin and transverse bands (a prominent component of the paranodal junctions), but exhibit an eversion of paranodal loops adjacent to the node, resulting in small but significant increases in nodal length [13, 16, 36, 61, 119]. The exact mechanisms involved in regulating changes in nodal length, however, are yet to be determined.

Intriguingly, altered neuronal activity can increase the length [78] and alter the position [42, 52] of the axon initial segment, which has molecular similarities to the node of Ranvier. However, it is not known whether similar molecular pathways underlie the increase of node length that occurs in pathology, nor whether alterations of node positions occur in the disorders mentioned above.

Subtle changes at the nodes which result in a failure of saltatory conduction may contribute to psychiatric disorders. Recent imaging studies in patients with schizophrenia [28, 96], bipolar disorder [2, 144], autism [4, 105] and personality disorder [86] have identified abnormalities in white matter integrity. Although the molecular basis for disease onset remains unknown, recent studies in schizophrenic and in bipolar patients have identified changes in nodal proteins. For example, microarray-based gene expression analysis across all brain regions of post-mortem brains from schizophrenia patients revealed a significant decrease in the expression of ankyrin G and neurofascin when compared to control tissue [121]. Furthermore, in the same study, analysis of the superior temporal gyrus, a region that has been shown to be altered in schizophrenia patients, found a significant downregulation of genes encoding for NrCAM and Nav1.6 in addition to neurofascin and ankyrin G [121]. Genome-wide association studies have found *ANK3* (the gene encoding ankyrin G) to be a susceptibility gene for schizophrenia, and associated identified single nucleotide polymorphisms (SNPs) with bipolar disorder [5, 10, 43, 121, 125, 127]. Moreover, a different study found an epistatic interaction between *ANK3* and KCNQ2 SNPs in bipolar disorder, i.e. the effect of a mutation in one gene was dependent on the mutation present in the other gene [72]. Given that these proteins are all found at high densities in the nodes of Ranvier as well as the AIS, it is possible that subtle abnormalities at these sites could affect action potential propagation and synchronicity of neuronal firing. *ANK3* has also been identified as a susceptibility gene in other disorders such as autism [14], attention-deficit/hyperactivity disorder (ADHD) [66, 80], intellectual disability [66] and epilepsy [70]. Although the nodes of Ranvier have not been directly investigated in these disorders, the importance of ankyrin G in node development and stability pinpoints this region as a promising new area to consider when investigating the molecular basis of psychiatric disorders.

## Molecular organisation of the paranode

The CNS node of Ranvier is defined by its flanking axo-oligodendroglial interactions which form the paranodes. Here, folds of uncompacted myelin interact directly with the neuronal axolemma, forming septate-like tight junctions. Although in vitro it has been shown that the paranodal junction is not a requisite for myelination [82], its formation is thought to be the first step in the development of nodes of Ranvier [141].

The paranode plays three important roles. Firstly, it acts as a diffusion barrier that separates the Nav channels in the node of Ranvier from the Kv1 channels in the juxtaparanodes. Studies on the AIS, which strongly resembles the node of Ranvier, in particular on the first paranodal junction at its distal end, have given insight into this. At this junction, the cytoplasmic proteins ankyrin-B, αII-spectrin and βII-spectrin define an intra-axonal boundary that precedes the presence of ankyrin G, which it localises in the developing AIS [157]. In addition, protein 4.1B is involved in the segregation of molecules between the AIS and the first myelin segment. Because of the similarity of the AIS to the node of Ranvier, it is generally assumed that the role of the paranode at the interface of the AIS and the first internode must be similar to its role when flanking the node of Ranvier.

Secondly, the paranode is required for “glueing” the myelin to the axon and ensuring a tight connection between the myelin and the axolemma. This is not only important for restricting membrane proteins to the node of Ranvier, but also for reducing current flow under the myelin sheath (see “Electrical principles for healthy node of Ranvier function” section above). The strong interaction between myelin and the axolemma is achieved by a complex of three proteins: axonal contactin-1 and contactin-associated protein 1 (Caspr1) and oligodendrocyte NF155. Knockout experiments in mice have shown that a deficiency in any of these proteins results in disruption of the axon–oligodendrocyte septate-like junctions which, in turn, causes axonal damage, slower nerve conduction and severe neurological defects, including ataxia and motor paresis, and death [13, 16, 49, 106, 129, 158]. Caspr1 and contactin-1 were first identified as neural adhesion molecules, which form a complex targeted to the paranodal junctions during myelination [39, 74, 103, 104, 116]. Shortly thereafter, NF155 was identified as the first glial component of the axo-glial paranodal junction [20, 143]. Contactin-1 and NF155 are similar in structure, both containing six immunoglobulin (Ig) domains and four fibronectin type III domains, but it is through the Ig domains that they are thought to interact with each other and with Caspr1. In turn, Caspr1 has a small cytoplasmic tail which contains a binding motif for the scaffold protein 4.1B, and this interaction is important for stabilisation of the Caspr1/contactin-1/NF155 complex at the paranode. The formation and maintenance of paranodal junctions are not only reliant on the Caspr1/contactin-1/NF155 complex, but also on GPI-anchored proteins, lipid raft-associated proteolipids and gangliosides, myelin galactolipids, as well as netrin and its receptor Dcc, which all play a role in the maintenance of the paranode, although the underlying mechanisms are poorly understood [56, 67, 68, 79, 99, 108, 124, 140].

Thirdly, using electron tomography Nans et al. [92] suggested a role for the paranode in directing protein traffic to the nodes. They identified an extensive network of filamentous linkers in the paranodal axoplasm connecting the juxtaparanode, the paranode and the node of Ranvier to each other, and showed that transport vesicles were tethered to the paranode by short filaments [92].

## Pathologies of the paranode

Given the complex molecular anatomy needed at the paranode to maintain the association between myelin and axons (Fig. 2), it is not surprising that it is a vulnerable structure in demyelinating disorders. Multiple sclerosis (MS) is a complex disease characterised by the demyelination of CNS axons affecting multiple parts of the brain and spinal cord, and leading to visual loss, paraesthesia, numbness, paralysis and other deficits. Although oligodendrocyte death plays a large part in the aetiology of MS, a number of studies have suggested that a disruption of the paranodal junctions may happen early in the onset of the disease. Post-mortem analyses of patients with multiple sclerosis show a disruption of the molecular organisation at the paranodes [24, 27, 64, 154] with decreased expression of Caspr and NF155 perhaps as a result of degradation [85, 154], and studies in an experimental model of MS (EAE) have shown that paranodal domain injury precedes the formation of internodal demyelinating lesions [47].

Several pathological conditions, including spinal cord injury [15, 69, 93, 130], demyelination caused by the *shiverer* mutation [131], multiple sclerosis and EAE [47, 64, 65], and glutamate application [48], result in paranodal disruption and a retraction of the myelin sheath. This produces an elongated appearance of the node of Ranvier as described above, and a movement of 4-AP-blockable Kv1 channels (which are normally found under the myelin sheath in the juxtaparanode, see below) closer to the elongated node (Fig. 3c–e) [64]. In some cases, the channels move out from under the myelin sheath so that they become more activated [15, 48, 69, 93, 131] (see “Electrical principles for healthy node of Ranvier function” section above), which will tend to oppose impulse propagation, especially during repetitive activity. Consistent with the loss of the barrier function of the paranode, preventing proteins moving from the node into the internode, post-mortem analyses of MS patients show a diffuse expression of Nav channels along the demyelinated axons in white matter lesions [26, 91]. In demyelinating regions, NF155 immunolabelling shows an increase in the length of the paranodal regions [64], suggesting a separation of the paranodal loops (Fig. 3d). It is therefore likely that disruption of the clustering of nodal proteins participates in the conduction and locomotor deficits occurring in MS patients. Similarly the alterations of the paranodal axo-glial junctions, and the exposure from under the myelin of Kv1 channels described above, might contribute to the conduction defects. In agreement with this, blockade of Kv1 channels using 4-AP (known clinically as fampridine) is a clinically approved approach to improve motor deficits and vision in patients with spinal cord injury and multiple sclerosis [15, 51, 63, 93, 126, 130] (reviewed by [57, 71]).

The mechanisms responsible for the alterations in the paranodes and nodal regions in MS have not yet been resolved, although new research is beginning to build a picture of the steps involved. MS is thought to be an autoimmune inflammatory disorder leading to demyelination and neurodegeneration. In agreement with this, antibodies to myelin have been identified in patients with MS [12, 115, 136, 137, 150, 155]. Interestingly, antibodies to the nodal and paranodal proteins NF155 and NF186 have also been observed in a subset of MS patients [41, 89], and these patients respond well to intravenous Ig injection and plasma exchange to remove the antibodies. A causal role for neurofascin antibodies in disrupting axonal conduction in a complement-dependent manner was corroborated by in vitro experiments applying the antibodies to hippocampal slice cultures [89]. Complement is a signal for attack by CNS microglia, and it has been shown that, independent of demyelinating lesions, there is a correlation between an increased microglial density and disruption of the paranodes in MS [65]. Furthermore, the elongation of paranodes observed in EAE mice can be blocked by preventing microglial activation with minocycline [65]. However, it is less clear whether microglial attack is involved in causing the paranodal disruption that occurs in spinal cord injury or with a raised extracellular glutamate concentration.

Intriguingly, disruption of the paranode has also been reported in ageing primates [60], with the movement of Kv1.2 channels from the juxtaparanode to the paranode where they are more likely to be activated as a result of better electrical coupling of the extra-axonal space to the extracellular space at the node. Conceivably, therefore, paranodal disruption could contribute to the cognitive decline that occurs in ageing.

## Molecular organisation of the juxtaparanode

Adjacent to the paranodal region lies the juxtaparanode (Fig. 1). This part of the axon is directly under the compact myelin, but does not extend for the entire length of the internode. Juxtaparanodes are enriched in Shaker-type Kv1 channels with Kv1.1 and Kv1.2 being the most predominant forms [112, 113, 148, 151, 152]. The importance of this cluster of Kv channels in mature myelinated axons, however, remains somewhat contested since, as reviewed above (see “Electrical principles for healthy node of Ranvier function” section), in normal conditions the application of 4-aminopyridine (to block Kv channels) to different axon tracts can have little effect on action potential propagation [45, 76] or can significantly broaden the action potential [32, 33]. Furthermore, at least in the PNS, whether Kv channels are clustered at the juxtaparanode or diffusely positioned along the internodes also seems to have little effect on the action potential propagation speed for single stimuli [107, 145]. This may be because the conduction speed depends primarily on the initial depolarizing phase of the action potential and less on its repolarization phase. Alternatively, it may reflect the fact that, even when these channels are correctly located at the juxtaparanode, they are little activated by the small voltage change they experience there. Despite this, Kv channels are thought to promote the fidelity of conduction by maintaining the internodal resting potential and preventing activation of any Nav channels present in the internodal axonal membrane. They thereby maintain the temporal precision of action potentials and dampen repetitive firing. During PNS node development, Kv1 channels prevent re-entrant excitation of the nodes [22, 50, 75, 77, 148].

The accumulation of Kv1 channels at the juxtaparanode is dependent on the cell adhesion molecules contactin-2 (known as TAG-1 in rodents) and Caspr2 [53, 107, 145]. Although deletion of contactin-2 or Caspr2 prevents the accumulation of Kv1 channels at juxtaparanodes, so that they are diffusely expressed along the internodes, as described above this has little effect on the speed of nerve conduction when tested in the PNS [107, 145]. Contactin-2 and Caspr2 are homologous to the paranodal proteins contactin-1 and Caspr1, and form a heteromeric complex on the axonal membrane. In contrast to the paranode (where axonal contactin-1/Caspr1 binds to oligodendroglial NF155), the oligodendroglial binding partner to the contactin-2/Caspr2 complex is contactin-2, which is also expressed on the myelin sheath. Similar to what happens at the paranodes, contactin-2 and Caspr2 are stabilised at the juxtaparanodes via an interaction with the scaffolding proteins 4.1B, ankyrin-B, αII-spectrin and βII-spectrin. All of these scaffolding proteins are also found at paranodes, suggesting that perhaps the segregation of paranodes and juxtaparanodes is in part accomplished by the different localisation of the glial adhesion molecules NF155 and contactin-2 [19, 23, 30, 38, 62, 100]. Also found at the juxtaparanodes is the desintegrin and metalloprotease ADAM22, which is closely associated with Kv1 channels [98]. ADAM22 recruits the MAGUK scaffold proteins PSD-93 and PSD-95 to the juxtaparanodes, but the exact function of this is unknown [98]. Intriguingly, in peripheral nerves ADAM22 is thought to play a role in controlling myelination via its interaction with a molecule secreted by Schwann cells, Lgi4 (leucine-rich glioma-inactivated 4) [102].

## Pathologies of the juxtaparanode

Mutations in *CNTN2* and *CNTNAP2* (the genes encoding contactin-2 and Caspr2, respectively) have been identified in autism spectrum disorder, epilepsy, Tourette’s syndrome, schizophrenia and ADHD [11, 40, 46, 90, 97, 135]. In the CNS, contactin-2 deletion leads to a loss of Kv1 clustering at juxtaparanodes, a reduction of internode length, and abnormalities of learning and memory, although it is unclear whether these are related to the paranodal and internodal alterations [123]. More recently it has been shown that, in addition to its role at the juxtaparanode, Caspr2 is expressed at synapses and involved in neural circuit assembly, and it has been suggested that it is a failure in this function that may underlie the effect of Caspr2 mutations on the development of autism [3].

Genetic mutations in Kv1 channels in humans, or depletion of the Kv1.1 gene in mice, result in hyperexcitability, episodic ataxia, myokymia and epilepsy [1, 17, 18, 25, 29, 83, 132, 156, 160], consistent with a role for Kv1 channels in limiting action potential generation. However, Kv1.1 and Kv1.2 channels can also be found in the AIS and axon terminals, and deletion of the Kv1.1 gene produces only a small prolongation of the compound action potential in mature nerves [132], making its loss at the juxtaparanode an unlikely explanation for the dramatic phenotypes observed in humans and mice. A more plausible explanation was provided for the PNS by Chiu and colleagues [22] who suggested that Kv channels expressed at the juxtaparanode of the last few internodes, before the synaptic terminal, play an important role in regulating neurotransmitter release.

Juxtaparanodal Kv channels are thought, however, to play an important role in demyelinating pathologies, where (as noted above) loss of the integrity of the paranodal axon–oligodendrocyte junction results in exposure of these channels from under the myelin. This will lead to them experiencing a larger voltage change during the action potential (see “Electrical principles for healthy node of Ranvier function” section above), and generating a larger outward membrane current, which contributes to the loss of action potential propagation. Blockade of these channels using 4-AP is a current treatment for relieving the conduction defects in multiple sclerosis and other demyelinating diseases [15, 51, 54, 63, 93, 126, 130] (reviewed by [57, 71]). However, the occurrence of seizures prevents administration of the high doses of 4-AP that are maximally effective at promoting action potential propagation in demyelinating axons [71], and it has been suggested that the channels being blocked to improve function are not in the demyelinating axons but at synaptic terminals [133].

Auto-antibodies to Kv1 channels have been found to contribute to Morvan’s syndrome and limbic encephalitis [73]. These frequently bind to juxtaparanodal K^+^ channels, suggesting that block of these channels contributes to the symptoms of neuromyotonia (spontaneous muscle fibre activity), confusion, anxiety, agitation, delirium or insomnia seen in these patients.

## Conclusions

Action potential propagation along myelinated axons is dependent on healthy nodes of Ranvier. A large and complex machinery has evolved to produce correctly functioning nodes of Ranvier by ensuring the correct targeting of Nav and Kv channels, the formation of tight junctions between paranodal loops and the neuronal axolemma, and the control of nodal length and diameter. Here, we have described the different domains which comprise the nodes of Ranvier and explained how changes at these sites underlie the variations in neuronal excitability observed in a number of neuropathologies. However, further work is still required to fully understand the molecular mechanisms that re-structure the node of Ranvier in disorders such as multiple sclerosis, ageing, spinal cord injury and neonatal hypoxia. In addition, proteins found at the node of Ranvier have been implicated in the pathology of psychiatric diseases such as schizophrenia, bipolar disorder, autism, personality disorder, ADHD and cognitive impairment, but it is still unclear whether the nodes of Ranvier and action potential propagation are indeed altered in patients with these disorders. Research into the basic mechanisms underlying the development, maintenance and disruption of nodes of Ranvier and paranodal junctions is likely to generate new therapeutic targets for neurological disorders of excitability.
